# The Potential Use of a Thin Film Gold Electrode Modified with Laccases for the Electrochemical Detection of Pyrethroid Metabolite 3-Phenoxybenzaldehyde

**DOI:** 10.3390/ma14081992

**Published:** 2021-04-15

**Authors:** Verónica Aglaeé Esquivel-Blanco, Gabriela Elizabeth Quintanilla-Villanueva, Juan Francisco Villarreal-Chiu, José Manuel Rodríguez-Delgado, Melissa Marlene Rodríguez-Delgado

**Affiliations:** 1 Universidad Autónoma de Nuevo León, Facultad de Ciencias Químicas, Laboratorio de Biotecnología, Av. Universidad S/N Ciudad Universitaria, San Nicolás de los Garza C.P. 66455, Nuevo León, Mexico; veronica.esquivelblnc@uanl.edu.mx (V.A.E.-B.); gabriela.quintanillavl@uanl.edu.mx (G.E.Q.-V.); juan.villarrealch@uanl.edu.mx (J.F.V.-C.); 2 Centro de Investigación en Biotecnología y Nanotecnología (CIByN), Facultad de Ciencias Químicas, Universidad Autónoma de Nuevo León. Parque de Investigación e Innovación Tecnológica, Km. 10 Autopista al Aeropuerto Internacional Mariano Escobedo, Apodaca C.P. 66629, Nuevo León, Mexico; 3 Tecnológico de Monterrey, School of Engineering and Sciences, Av. Eugenio Garza Sada Sur No. 2501, Col. Tecnológico, Monterrey, C.P. 64849, Nuevo León, Mexico

**Keywords:** thin-film, gold electrode, electrochemical biosensor, enzyme, laccase, pyrethroid metabolite, 3-Phenoxybenzaldehyde

## Abstract

There is increasing interest in developing portable technologies to detect human health threats through hybrid materials that integrate specific bioreceptors. This work proposes an electrochemical approach for detecting 3-Phenoxybenzaldehyde (3-PBD), a biomarker for monitoring human exposure to pyrethroid pesticides. The biosensor uses laccase enzymes as an alternative recognition element by direct oxidation of 3-PBD catalysts by the enzyme onto thin-film gold electrodes. The thin-film gold electrode modified by the immobilized laccase was characterized by Fourier-transform infrared spectrometry and scanning electron microscopy. The detection method’s electrochemical parameters were established, obtaining a linear range of 5 t 50 μM, the limit of detection, and quantification of 0.061 and 2.02 μM, respectively. The proposed biosensor’s analytical performance meets the concentration of pyrethroids detected in natural environments, reflecting its potential as an alternative analytical tool for monitoring the pyrethroid insecticide’s presence.

## 1. Introduction

Pyrethroids are synthetic pesticides chemically based on the natural pyrethrins found in Chrysanthemum sp. flowers [1]. This primary class of insecticides has been extensively used without restrictions over the past decades as they have relatively low toxicity to mammals and broad-spectrum resistance to pests [2]. The excessive use of these pesticides has caused their accumulation in products for human consumption, in addition to a wide diversity of natural environments [3,4]. They tend to negatively impact the biochemistry of aquatic species [5,6] and beneficial insects such as honeybees and ground beetles [7,8] as pyrethroids act as sodium channel toxins that alter the gating of neuronal cell channels, causing insects’ paralysis and death [9].

Although these insecticides are considered to be of low toxicity to humans, some studies have demonstrated that pyrethroids can suppress the immune system [10], disrupt the endocrine system [11], and cause carcinogenesis [12]. In this sense, Zepeda-Arce et al. (2017) associated occupational exposure to pyrethroid pesticides with oxidative stress in cells, causing DNA damage [13]. Furthermore, it is suggested that exposure to the general population occurs through pyrethroid residues present in food, drinking water, or by contact with particles remaining in the air after residual use, through inhalation and dermal contact [14]. Fortunately, the short half-lives of pyrethroids avoid more significant toxicity in the population; however, this complicates their direct detection [14]. Under environmental conditions, the carboxyl ester bond of pyrethroids (mainly β-cypermethrin) is hydrolysed to produce 3-Phenoxybenzaldehyde (3-PBD) as the primary metabolite [15]. However, 3-PBD is a toxic compound that has been classified as an endocrine disruptor. It is more mobile than its pyrethroid precursors, causing widespread contamination [16,17]. Thus, the detection of pyrethroid metabolites such as 3-PDB is currently used as a biomarker to assess the level of pesticide exposure or residual contamination [18].

To date, the detection of pyrethroids and their metabolites is commonly performed by chromatographic methods. Such methods include liquid chromatography coupled with mass spectrometry [19], gas chromatography and electron capture detection [20], high-performance liquid chromatography (HPLC), and supercritical fluid chromatography (SFC) [21]. However, the high operating costs, time-consuming sample pre-treatments, and laboratory-based instrumentation remain significant limitations on these routine methods. Therefore, it is imperative to develop alternative methods for the direct detection of pyrethroid insecticides. In this context, different bio-coatings or modified surfaces have been studied for their application in the development of sensitive and specific receptors for the detection of pyrethroids and their metabolites. For example, Ye et al. 2018 studied a molecularly imprinted polymer (MIP) as a recognition element for the detection of 3-PBD and coupled it with a colourimetric method using potassium permanganate [22]. In the study, the MIP layer was coated via a sol-gel process with 3-Aminopropyltriethoxysilane (APTES) and Phenyltrimethoxysilane (PTES). PTES possessed the ability to interact with 3-PBD by hydrogen bonding and π-π staking interaction, showing the high affinity and absorption capacity towards the target molecule [22]. Other studies have taken advantage of the oxidated form of 3-Phenoxybenzaldehyde (3-Phenoxybenzoic acid) to detect the metabolite through competitive immunoassay by using antigen–antibody interaction onto modified surfaces [23,24]. Despite the high sensitivity and selective detection of immunoassays, this methodology is currently limited by the monoclonal antibodies’ vulnerable stability, increasing precautions during the manipulation steps to detect the pyrethroid metabolite [22].

On the other hand, recent advances in hybrid thin-films, which integrate a bioreceptor for target molecules and signal transduction, have attracted much interest in the construction of electrochemical sensors [25]. In this sense, laccase enzymes, which are produced by a wide diversity of plants and microorganisms [26,27,28], have been widely used as bioreceptors in biosensors due to their high stability and wide range of catalytic oxidation of organic compounds [29] in the presence of oxygen [30]. In particular, laccase stands out among other enzymes (such as acetylcholinesterase, AChE) by its ability to catalyse the biodegradation of pyrethroids (e.g., cypermethrin, imiprothrin) [31,32,33] without suffering inhibition [34]. Therefore, this work explores the use of laccase enzymes as an alternative recognition element for the electrochemical detection of the pyrethroid metabolite 3-PDB by its direct oxidation on thin-film gold electrodes. For this, the thin-film surface of the gold electrode modified by the immobilized laccase was characterized, and the detection method’s electrochemical parameters (e.g., the limit of detection, the limit of quantification, and working range) were established.

## 2. Materials and Methods

### 2.1. Reagents

The laccase enzymes from *Rhus vernicifera* and salts employed in buffer solutions were purchased from Sigma-Aldrich (St. Louis, MO, USA). The chemical compounds 3-Phenoxybenzaldehyde, 16-mercaptohexadecanoic acid (MHDA), 11-mercaptoundecanol (MUD), ethanolamine hydrochloride, *N*-hydroxysuccinimide (NHS), and 1-thyl-3-(3-dimethylamino-ropyl) carbodiimide hydrochloride (EDC) were supplied from Sigma-Aldrich (St. Louis, MO, USA). The stock solutions of 3-Phenoxybenzaldehyde (100 mg·mL^−1^) were prepared in ethanol:water (90:10, % V/V), and from them, dilutions were prepared in phosphate buffer solution (PBS), pH 7.3 (0.1 M). Chrome and gold pellets (99.999%) were purchased from Kurt J. Lesker Co. (Clairton, PA, USA) and employed in the thin-film process’s evaporation.

### 2.2. Instrumentation

The electrochemical analyses were performed using a three-electrode scheme, including a laccase-gold working electrode (Lac-Au electrode), a platinum counter electrode, and Ag/AgCl (3.0 mol L^−1^·KCl) as the reference electrode at 25 °C, to which all potentials are referred. Cyclic voltammetric analyses were conducted from −0.8 to 0.4 V with a scanning rate of 0.1 V·s^−1^, using a workstation CHI700E (CH Instruments, Inc.; Bee Cave, TX, USA). Electrochemical impedance spectroscopy (EIS) measurements were performed with a frequency range from 1 Hz to 100 kHz and alternate current amplitude set at 10 mV, recorded in the CHI700E (CH Instruments, Inc.; Bee Cave, TX, USA) potentiostat. The scanning electron microscope (SEM-JSM-7800F, JEOL Ltd., Tokyo, Japan) was employed to examine the gold thin-film electrode’s surface before and after laccase immobilization. Finally, the Lac-Au electrode was evaluated by infrared analysis using a Spectrum 100 FTIR spectrometer (PerkinElmer Inc.; Waltham, MA, USA) in the region between 4000 and 650 cm^−1^ (resolution of 50 scans at 4 cm^−1^), using a KBr window for solutions.

### 2.3. Fabrication of Lac-Au Electrode

The working electrode fabrication consisted of a step-by-step process, where glass substrates (1 cm^2^) previously coated with a chromium layer were subjected to a gold thin-film deposition, following the method described by Luna-Moreno [35]. Since gold has a very poor adherence to glass, a chromium layer between the glass and the gold is highly recommended to improve the attachment [35]. Briefly, a first chromium layer was evaporated up to 3 nm thickness by electron gun evaporation using a High Vacuum Coating Plant BA510 (Balzers High Vacuum Corp., Santa Ana, CA, USA) with a rate of 1.0 Å·s^−1^ and an atmosphere of 8 × 10^−6^ mbar. A gold film of 50 nm was then deposited by thermal evaporation at the rate of 5 Å·s^−1^ and 8 × 10^−6^ mbar. The thin film’s thickness was evaluated employing a thickness monitor of quartz crystal microbalance (XTC/2 Depositions Controllers Leybold Inficon quartz monitor, San Jose, CA, USA). Before modification, the bare gold electrode was carefully polished with 0.05 mm alumina slurry, followed by successively sonicating it in ultrapure water and absolute ethanol (3 min in each solvent). Then, it was dried at room temperature.

Afterward, the clean electrode was immersed overnight in a solution of alkanethiols MHDA: MUD (250 µM in ethanol) [35]. The sulphur atoms of the alkanethiols bind covalently to the gold, allowing the free carboxylic groups to anchor the enzyme in further immobilization at the end of the molecule. Once functionalized, the carboxylic groups on the electrode’s surface are activated by the EDC/NHS crosslinkers (0.2 M/0.05 M) solution prepared in MES buffer (100 mM, 500 mM NaCl, pH 5.0) [35]. Finally, an enzymatic solution of 100 U·mg^−1^ is cast onto the gold-coated electrode, allowing the laccase immobilization through the formation of an amide bond between the lysines of the enzyme and the activated carboxylic groups of alkanethiols.

### 2.4. Laccase Enzyme Activity

The laccase activity was evaluated through the spectrophotometric assay adapted from Zhang et al. 2018 [36], where 200 µL of the enzyme is added to a solution containing 10 mM of ABTS (2,2’-azino-bis(3-ethylbenzothiazoline-6-sulfonic acid)) in 0.1 M sodium acetate buffer, pH 4.5. The ABTS substrate’s oxidation is recorded at 420 nm in a UV-Vis spectrophotometer (Cary 50, Varian Inc., Palo Alto, CA, USA). The activity units (U) are expressed as a function of the amount of enzyme necessary to produce 1 µM·min^−1^ of product.

### 2.5. Electrochemical Analysis

The electrochemical measurements were performed on a solution prepared with an appropriate amount of 3-Phenoxybenzaldehyde (3-PBD) in phosphate buffer solution (PBS), pH 7.3 (0.1 M). The current increments in the cyclic voltammetric analysis from 5 µM to 50 µM were recorded. The linear fitting of the 3-PBD concentration-dependent current response curve was conducted to calculate the method’s detection sensitivity. The detection limit was evaluated as three times the standard deviation of the baseline, while the limit of quantitation was 10 times the standard deviation.

## 3. Results and Discussion

### 3.1. Fabrication of Lac-Au Electrode

**Figure 1:**
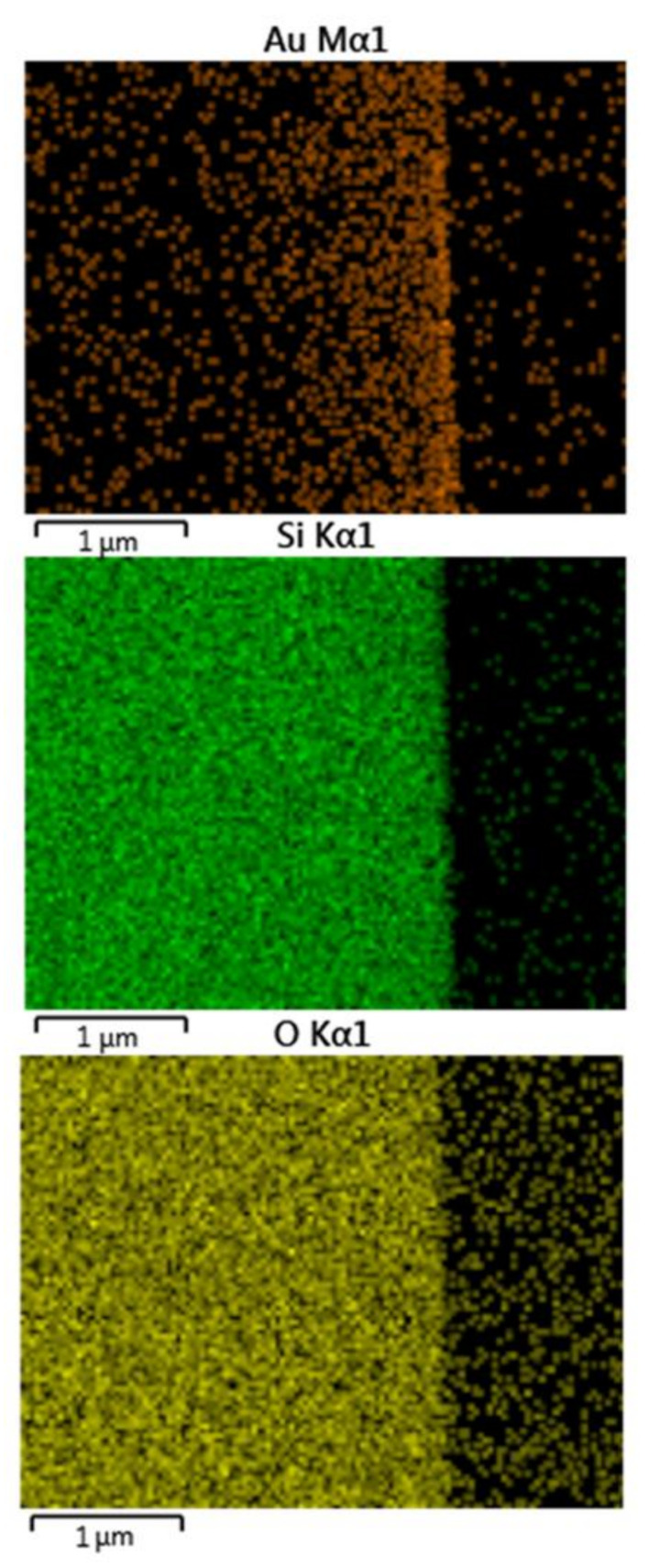
Elemental mapping images from the cross-sectioned gold-coated substrate.

The Lac-Au working electrodes used in this study were fabricated in a step-by-step process, where glass substrates (1 cm^2^) were initially covered with 3 nm of chromium, which was used as an adherent to improve the attachment of gold to the glass substrate. Once the chromium layer was formed, a thin film of gold was deposited by thermal evaporation. The gold layer deposited on the glass substrate was observed in the elemental mapping images from the cross-sectioned material (Figure 1).

**Figure 2:**
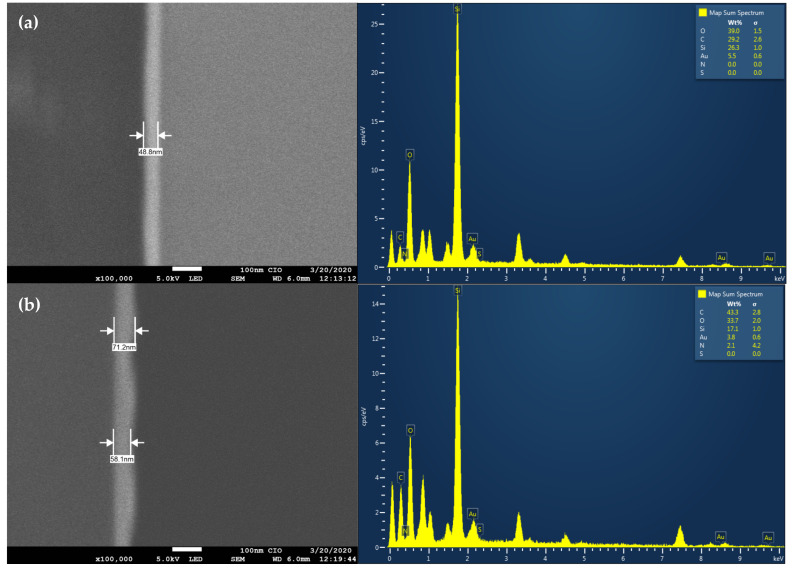
Cross-sectioned SEM images of the gold thin film before (**a**) and after (**b**) laccase immobilization.

SEM images of the cross-sectioned gold-coated substrate exhibited that the deposition of the gold thin film was homogeneously distributed across the substrate, measuring around 49 nm (Figure 2a). The thin-film’s surface exhibited a smooth texture, which was ideal for the following enzyme immobilization process—this process initiated the functionalization of the gold-coated surface with alkanethiols. After overnight incubation, the functionalized carboxylic groups were activated through the cross-linkers EDC/NHS, forming an amide bond that anchored the laccases. Once the immobilization process concluded, SEM micrographs corroborated the electrode surface’s modifications. In this sense, Figure 2b shows the layer’s irregularity formed onto the gold film, suggesting the enzymes’ addition. Before the immobilization process, the gold film had a thickness of around 49 nm. After the immobilization process, the layer presented a width ranging from 58 to 71 nm.

**Figure 3:**
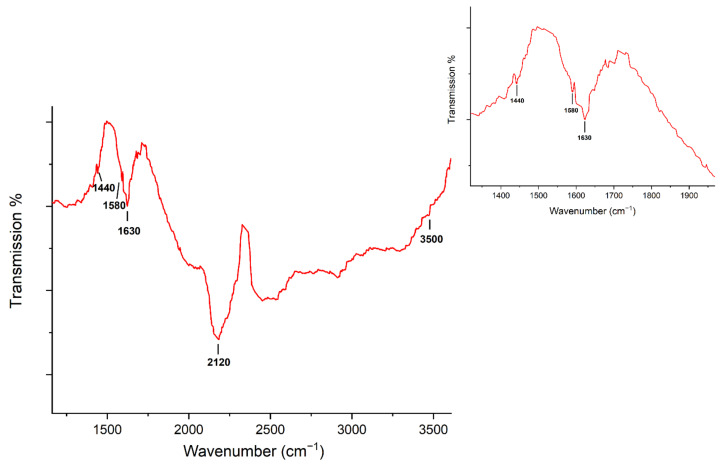
FTIR spectrum of gold thin film immobilized with laccase (Inset: Zoom of FTIR region from 1300 to 1700 cm^−1^).

The irregularity of the electrode surface could be attributed to the formation of enzyme–enzyme conjugates, which can be formed during the immobilization process due to excessive cross-linker concentration that activates the carboxylic groups within enzymes as well as those from the alkanethiols [37]. These enzyme–enzyme conjugates are formed by the cross-linking between the side chains of the proteins’ amino acids by forming covalent bonds between them [37]. In particular, laccase enzymes are rich in amine groups from lysine residues and carboxyl groups from glutamic and aspartic acid, which are capable of interacting as suggested by Addorisio et al. [38]. The FTIR spectroscopy characterization of the immobilized gold surface (Figure 3) exhibited the characteristic bands of amide I at 1580 cm^−1^ (NH, bending) and 3500 cm^−1^ (NH, stretching), amide II at 1630 cm^−1^ (C=O, stretching), and the symmetric stretching of carboxylates vibration at 1440 cm^−1^ [39]. According to Schartner et al., these bands’ presence confirms the proteins’ immobilization through lysine side chains [40]. On the other hand, an additional signal was observed at 2120 cm^−1^, commonly attributed to the carbodiimide group (N=C=N, stretching) of the EDC cross-linker. This band’s presence suggests that some functionalized surface sites remained activated and without enzymes [41]. Once the immobilization process concluded, the enzymatic activity of immobilized laccase was measured and compared to the initial activity of 100 U·mg^−1^, obtaining a 34% loss in the free enzymes’ oxidation activity. The decay in the enzymatic activity after an immobilization process is well known and has been attributed to non-oriented covalent immobilization of the enzyme that blocks the molecule’s active site [42]. The loss of enzymatic activity obtained in our work was similar to that reported by Fan et al. 2017, where an esterase lost 40% of its activity after its immobilization on silica, using a similar method based on the formation of covalent bonds [43].

**Figure 4:**
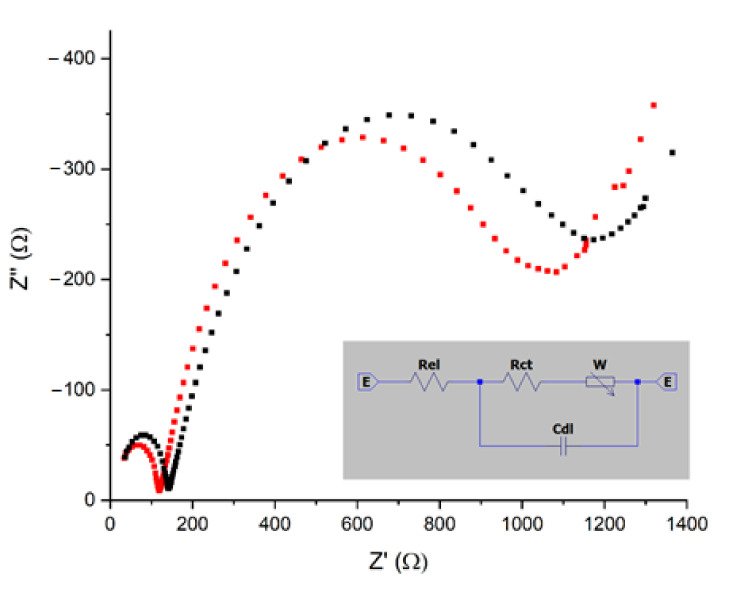
Electrochemical impedance spectroscopy analysis on the bare gold electrode (red dots) and gold electrode modified with laccase (black dots). Inset: equivalent circuit of the Lac-Au working electrode.

Once the electrode fabrication was completed, electrochemical impedance spectroscopy (EIS) was performed to analyse the conductivity of the modified electrode. Figure 4 shows the impedance features of both bare and immobilized electrodes. From this information, an equivalent circuit was simulated for the working electrode (Figure 3 inset). The equivalent circuit consisted of an electrolyte solution resistance (Rel) parallel with a double layer capacitance Cdl and a charge electron-transfer resistance Rct, which is in series with Warburg impedance (W), which indicates the diffusion of the ionic species through the diffusion layer [44]. The bulk properties of the electrolyte solution and diffusion of the redox species in solution are represented in Rel and W, which were affected by the enzyme immobilization at the electrode surface. This array increments the resistance from 20 to 30 Ω and diffusion from 0.0012 to 0.0015 S·*√*s* (see Table 1).

On the other hand, the parallel combination of Rct and Cdl indicates the insulating and the dielectric characteristics of the electrode/electrolyte interface, represented as a semicircle in the Nyquist plots (Figure 4), and the charge electron-transfer resistance Rct was obtained by measuring the diameter of the semicircle in the impedance spectrum [44]. The Rct for a bare Au electrode was estimated to be 100 Ω and increased slightly to 112 when the electrode was modified with laccase, generating an insulating layer on the electrode surface that barrier the interfacial electron transfer. This result suggested the assembly of the enzymes on the gold electrode’s surface.

### 3.2. Electrochemical Measurements

**Figure 5:**
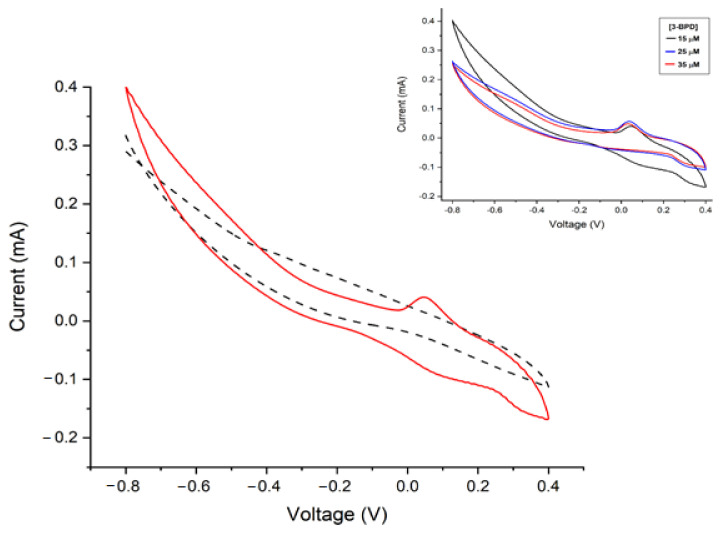
Voltammetric profile of Lac-Au working electrode (black dotted line: scan in phosphate buffer solution (PBS); red line: measurement in PBS with 3-PBD). Inset: voltammetric profile in PBS with 15, 25, and 35 µM of 3-PBD.

Once the laccase enzymes were immobilized on the gold electrodes, these Lac-Au working electrodes were tested for their use as a potential recognition element in developing a sensitive biosensor to detect the pyrethroid metabolite 3-Phenoxybenzaldehyde (3-PBD). The biosensor’s cyclic voltammetry behaviour against 3-PBD in a phosphate buffer solution (pH 7.3) was recorded from −0.8 to 0.4 V with a scanning rate of 0.1 V·s^−1^. As shown in Figure 5, an anodic peak was detected at −0.02 V, suggesting the oxidation of 3-PBD. This process may be attributed to laccase activity on the oxidation of the aldehyde to carboxylic acid in the presence of oxygen [45]. Nevertheless, more studies need to be carried to characterize the oxidation product identity. Furthermore, no reduction current was observed during the experiment, suggesting the irreversibility of the 3-PBD reaction.

**Figure 6:**
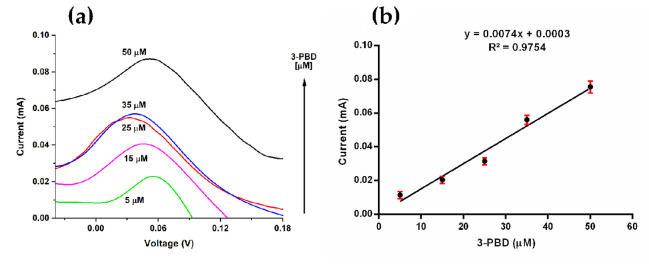
(**a**) Cyclic voltammetry of 5, 15, 25, 35, and 50 µM in 0.1 M PBS (pH 7.3) with the scan rate of 0.1 V·s^−1^ and (**b**) Calibration curve of oxidation peak current versus concentration.

When 3-PBD was added to the working solution, the current of the oxidation peak increased linearly and proportionally to the concentration ranging from 5 to 50 µM (Figure 6a), following the equation I_(mA)_ = 7.4 × 10^−3^ *c* + 3.0 × 10^−4^ [µM] with a correlation coefficient of 0.975 (Figure 6b).

Our electrochemical setup exhibited a sensitivity of 7.4 × 10^−3^ mA/µM, a limit of detection (LOD) and quantification (LOQ) of 0.061 ± 0.002 and 2.02 ± 0.001 µM, respectively. These results suggest that our device can be used to detect pyrethroids in different natural samples, such as rivers, fish, and sediments [3] since the maximum and minimum concentrations reported to date were 2.38 mM in fish and 0.439 µM in drinking water from India [3]. This method’s analytical parameters are comparable with those obtained employing different biosensing strategies, as presented in Table 2.

Overall, laccase proved to be feasible to monitor the presence of the biomarker 3-PBD in a concentration range that corresponds to the natural occurrence of pyrethroid pesticides. However, despite promising results, it is essential to continue developing this technology to confirm the method’s robustness and determine the selective detection of 3-BD. Furthermore, future work should focus on validation testing on real samples.

## 4. Conclusions

In summary, a hybrid thin-film consisting of laccase immobilized onto a gold surface was designed as an electrochemical approach for the detection of metabolite pyrethroid 3-Phenoxybenzaldehyde. Under the tested conditions, the Lac-Au electrode was applied for detecting 3-PBD with a linear range of 5 μM to 50 μM and a low limit of detection of 0.061 μM. The analytical performance of the approach method proposed here meets the concentration of pyrethroids detected in real environments, offering a potential alternative to monitoring the pyrethroid insecticides’ presence. The gathered results shed light on the possible use of the laccase enzyme as a recognition element prospect in the field for analysis of pyrethroids exposure. Nevertheless, the provided method is treated as an initial stage towards further investigations within the optimization and validation of the electrochemical analysis.

## Figures and Tables

**Table 1:** Comparison of equivalent circuit components of bare and immobilized gold electrode.

Electrode	Rel (Ω)	Rct (Ω)	Cdl (F)	W (S·*√*s*)
Bare Au	20	100	4.5 × 10^−9^	0.0012
Lac-Au	30	112	5.0 × 10^−9^	0.0015

**Table 2:** Analytical strategies for detection of pyrethroids metabolites.

Strategies	Element of Recognition	Analyte	Limit of Detection (µM)	Reference
Colorimetric	Molecularly imprinted polymers	3-PBD	0.262	[22]
Electrochemical	Laccase enzyme	3-PBD	0.061	This work
Photoluminescence	Mn-doped ZnS quantum dots	3-PBA	0.117	[46]
Microarray immunoassay	antibodies	3-PBA	0.007	[24]
Colloidal gold-based lateral flow immunoassay	antibodies	3-PBA	5.04	[47]

3-PBD: 3-Phenoxybenzaldehyde; 3-PBA: 3-Phenoxybenzoic acid.

## Data Availability

The data presented in this study are available on request from the corresponding author.
